# Muscle oxygen dynamics in elite climbers during finger-hang tests at varying intensities

**DOI:** 10.1038/s41598-020-60029-y

**Published:** 2020-02-20

**Authors:** Andri M. Feldmann, Daniel Erlacher, Sandro Pfister, Remo Lehmann

**Affiliations:** University of Bern, Institute of Sport Science, Bremgartenstrasse 145, 3012 Bern, Switzerland

**Keywords:** Metabolism, Predictive markers

## Abstract

The aim of this study was to measure muscle oxygen saturation (SmO_2_) dynamics during a climbing specific task until failure in varying conditions. Our prediction was that SmO_2_ should be a good marker to predict task failure. Eleven elite level climbers performed a finger-hang test on a 23 mm wooden rung under four different weighted conditions, 1. body weight (BW), 2. body weight +20% (BW +20), 3. body weight −20% (BW −20) and 4. body weight −40% (BW −40), maintaining half crimp grip until voluntary exhaustion. During each trial SmO_2_ and time to task failure (TTF) were measured. TTF was then compared to the minimally attainable value of SmO_2_ (SmO_2_min) and time to SmO_2_min (TTmin). There is a considerable degree of agreement between attainable SmO_2_min at high intensity conditions (*M*_*BW*_ = 21.6% ± 6.4; *M*_*BW*__+20_ = 24.0% ± 7.0; *M*_*BW−20*_ = 23.0% ± 7.3). Bland-Altman plot with an a priori set equivalency interval of ±5% indicate that these conditions are statistically not different (*M*_*BW-BW* + 20_ = −2.4%, 95% CI [1.4, −6.2]; *M*_*BW−Bw−20*_ = −1.3, 95% CI [2.5, −5.1]). The fourth and lowest intensity condition (*M*_*BW −40*_ = 32.4% ± 8.8) was statistically different and not equivalent (*M*_*BW-BW −40*_ = −8.8%, 95% CI [−5.0, −12.6]). The same agreement was found between TTF and TTmin for the high intensity conditions plotted via Bland-Altman. While the rate with which oxygen was extracted and utilised changed with the conditions, the attainable SmO_2_min remained constant at high intensity conditions and was related to TTF.

## Introduction

Failure in elite sport climbing is often associated with a fall as a result of the inability to maintain the required force of isometric muscle contraction following repeated efforts^1^. For this reason, isometric force production and maintenance in sport climbing has been a growing area of interest for sport scientists and exercise physiologists. This is further underscored by the fact that traditional forms of non-sport specific athletic testing such as VO_2_max assessments or blood lactate analysis have been unsuccessful in determining sport specific fitness^2–5^. This is likely due to the highly sport specific muscular demand and muscular development that is required for sport climbing disciplines^3,6,7^. Near-infrared spectroscopy (NIRS) as a tool to assess local oxidative capacity has proven to be useful^8–11^ and has rightfully garnered more interest to investigate local muscle physiological dynamics due to its non-invasive and simple application to *in vivo* and *in situ* diagnostics. NIRS has been compared to measurements of phosphorus magnetic resonance spectroscopy (P-MRS) to assess PCr dynamics with a good degree of agreement^10^, as well as in comparison with *in situ* high-resolution respirometry to assess mitochondrial respiratory capacity^11^. This capability has allowed a highly focused pursuit in the understanding of local muscular capacity for performance, especially in this case the importance of local oxidative capacity in sport climbing^12–16^. A greater local oxidative capacity has shown to enhance sport specific strength and endurance when comparing novice and expert climbers^13^, with a considerable degree of predictive power to determine climbing level; linear regression model predicting Red-point grade (IRCRA Scale) from tissue oxygen resaturation rate: y = −0.351 + 14.121, R^2^ = 0.24^13^. Numerous studies look both at isometric holds and repeated or intermittent isometric holding as performance limiting components and their effect on NIRS dynamics^12,15,17^. Specifically, sustained isometric holds on static gripping tasks have shown to result in NIRS dynamics that indicate loss of force production^16^. During a finger-hang test, similar to the one conducted in this experiment, Balas *et al*.^18^ identified that this test, from a series of tests, explained the greatest portion of the performance variance (70%) in a climbing population. Applying this knowledge to simple *in vivo* and *in situ* tests during isometric finger-hangs, it is possible that NIRS dynamics could be used to assess task failure during measurement situations, which in turn could be a useful tool for both training and competition.

For these predictions the minimally attainable muscle oxygen saturation (SmO_2_min) can be used as a pragmatic surrogate for oxidative capacity^8,14,16,19^. If oxidative capacity is an individual, but constant volume, then the rate of muscle deoxygenation should increase but SmO_2_min would remain constant near to the point of task failure. This highlights two research hypothesis; 1. during a sport specific performance task SmO_2_min remains constant over varying workloads; and 2. time to SmO_2_min (TTmin) is equal to time to task failure (TTF).

## Methods

### Experimental approach to the problem

A repeated measures design was applied to assess muscle oxygen (SmO_2_) dynamics as measured by NIRS during sport specific finger-hang tests. The purpose of this design was to elicit a force-duration relationship in order to assess physiological and performance related changes over varying intensities. A classical performance-duration relationship is curvilinear flattening to an asymptote with a curve constant. The curve constant represents something in the form of work capacity over this asymptote. SmO_2_min reflects this work capacity and the change in energetics and metabolite accumulation associated with it^20^. SmO_2_min is the primary variable assessed in this study due to the relationship between NIRS signal range and oxidative capacity discussed earlier. Therefore, each participant partook in four trials of the finger-hang test under four separate conditions; body weight (BW), body weight +20% (BW +20%), body weight −20% (BW −20%) and body weight −40% (BW −40%). Each trial was separated by at least 20  minutes of rest, similar to finger-hang test protocols in other studies^12,15^. All participants started with the BW trial and then the following three trials were in randomized order to eliminate a serial effect. The BW trial was used as the reference trial for comparison against the remaining three trials. Adipose tissue thickness at the sensor location was deemed unproblematic considering the source-detector distance of 25  mm (penetration depth of 12.5  mm)^21,22^. All participants were asked to refrain from strenuous physical activity 24  hours prior to the experiment, to refrain from alcohol consumption, caffeine consumption and smoking 24  hours prior to the experiment and to maintain individual diet routine.

### Participants

The participants were all male elite level climbers (n = 11; ±SD age 24 ± 4.0 years; height 172.9 ± 7.1  cm; weight 65.7 ± 2.5  kg; climbing level: redpoint 27 ± 2.5 IRCRA^23^). Climbing level was assessed through a questionnaire and was defined by self-reported achievements within the last 3 months. The select population was, therefore well prepared for the physical tasks presented by the data collection minimizing potential risk of injury. All participants were in good health, non-smokers and unmedicated. The participants were informed of the study design and the physical tasks ahead of time and written informed consent was obtained in advance. The study was carried out in accordance with the 1964 Declaration of Helsinki. The University of Bern faculty of Human Sciences Ethics Commission approved the protocol (Nr. 2018-03-00009).

### Warm-up

All participants conducted a standard warm-up. First, a 3-minute bike ride with 75 watts resistance at 75–80  rpm. Then two sets of 10 repetitions self-selected (5–10  kg) forearm curls and two sets of 10 repetitions of finger extensions using a TheraBand, followed by self-selected arm-hand-finger mobilisation exercises. Finally, three minutes of self-selected trials on the hang-board to familiarise themselves with the equipment. Participants were informed to avoid over excretion during warm-up. Considering the expertise and fitness level of the participants this was considered unproblematic.

### Finger-hang test

A finger-hang test was selected for this study as Balas *et al*.^18^ showed that in their model the finger-hang test alone explained approximately 70% of the variance in climbing performance. The finger-hang test was conducted on a commercially available hang-board (AIX, CZ). Participants were informed of the procedure and were instructed to maintain their grip on the hang board with both hands for as long as possible. Minor readjustments during a trial were allowed, as long as both hands remained on the rung and in the same effective position. The hand position applied by all participants was a half crimp; the thumb was not used. A 23  mm rung depth was used for all the trails, following the procedure of a series of previous publication^17,24^. For every trial the participants were allowed to apply commercially available climbing chalk (magnesium carbonate). Prior to every trial the holding rung was cleaned and cleared of excess chalk. The participants were asked to take a standing position with their arms down and to the side in a natural posture in front of the hang-board where base-line physiological measurements were recorded for 30  seconds. The participants were then asked to prepare themselves on the holding rung. On command the participants lifted their legs and took on the hanging position and the task-time measurement started. The task was considered failed and the timer stopped if either a hand left the hang-board or when a foot returned to the floor. After failure two minutes of data recording followed where the participant again took a natural standing position with arms down and to the side. The same procedure was followed for all the trials and conditions. For the +20%BW condition the participants were equipped with a commercially available climbing harness and the appropriate amount of weight was added using a carabiner and a sling around the belay loop. For the −20%BW and −40%BW conditions a single-wheel pulley system was setup directly behind the hang-board. The participant was attached to one end of the pulley via the climbing harness (belay loop) and the appropriate amount of weight was added to the second end. The pulley system was evaluated and adjusted for mechanical advantage and the appropriate weight was used.

### Near-infrared spectroscopy

NIRS sensors were placed on the forearm flexor digitorum profundus (FDP) muscles, which can be palpated on the anterior side of the forearm one-third between the medial epicondyle of the humerus and the styloid process of the ulna; discussed in point-counterpoint discussion^25,26^ and considering the importance and relevance of the NIRS probe placement the clear guidelines by Fryer *et al*.^13^ was used. The flexor groups, including the FDP have their primary function in finger flexion for the pre-determined task^27,28^. The sensors were fixed in place using medical adhesive tape (Hypafix; BSN Medical, DE) and were then covered with the compatible commercially available light shield to eliminate possible ambient light intrusion. A commercially available continuous wave NIRS device was used (Moxy Monitor; Fortiori Designs LLC, US) to measure muscle oxygenation. The device uses four wave lengths (680,720,760 and 800  nm) to assess absorbency of oxyhemoglobin (O_2_Hb) and oxymyoglobin (O_2_Mb) and deoxyhemoglobin (HHb) and deoxymyoglobin (HMb), via modified Beer-Lambert resulting in a concentration of SmO_2_ as a percent in the following equation:

$$Sm{O}_{2}=\frac{Hb+{O}_{2}Mb}{({O}_{2}Hb+{O}_{2}Mb)+(HHb+HMb)}$$

Myoglobin and hemoglobin cannot be differentiated using NIRS, and therefore as shown in the equation above, are always evaluated together^29^. SmO_2_ is a ratio of relative O_2_Hb + O_2_Mb and relative HHb + HMb on an absolute scale of 0–100%^21^. The device detectors are spaced at 12.5  mm and 25  mm from the emitter. Sampling rate was set at default mode which samples the four wave lengths over 80 cycles for an averaged output every two seconds (0.5  Hz) and gathered using the SWINCO NIRS software (Swinco AG, CH).

### Statisical analyses

**Figure 1:**
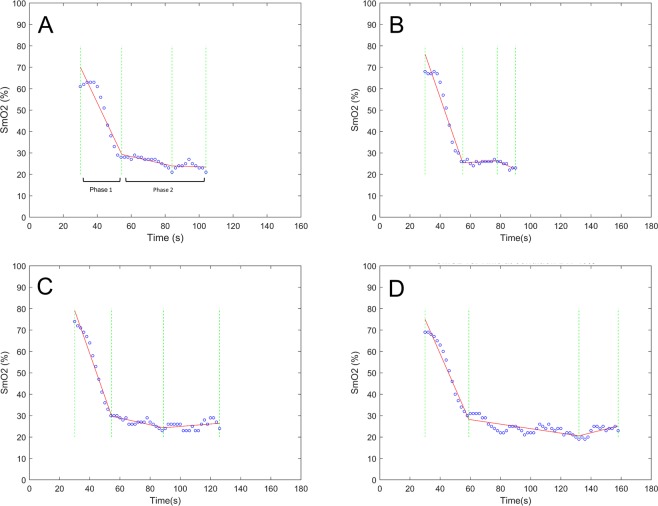
Piecewise regression analysis identifying SmO_2_min and TTmin from a select participant for all four trials; (**A**) body weight; (**B**) body weight +20%; (**C**) body weight −20%; (**D**) body weight −40%. Participants exhibited the expected pattern of two phases of decreasing SmO_2_ slopes. In order to determine the SmO_2_min point a four knots solution was applied to identify start and end, as well as shift from phase 1 to phase 2 and then the minimum point in phase 2.

In order to assess the agreement between trials Bland-Altman plots were constructed to present the data. As the goal of the statistical analyses was to find agreement rather than difference Bland-Altman plots were specifically chosen to address the question, as agreement is not within the scope of traditional inference statistics. Where relevant significance level was set at 0.05. The four conditions were compared for agreement as follows: SmO_2_min BW against SmO_2_min of all the remaining conditions and TTF against TTmin for all conditions. Upper and lower limits of agreement were set at 1.96  SD with recommended 95% confidence intervals (CI)^30^. Additionally, a priori equivalency intervals (EI) were set at the recommended minimally detectable change of  ±5%^17^ in agreement with other device specific recommendations^31^ for the SmO_2_min analysis. In order to determine SmO_2_min a least squares piecewise regression analysis was conducted to find the break point at the minimally attainable SmO_2_ (Fig. 1). Similar to the results of isometric holds in other data collections^24^ two clear phases of deoxygenation can be seen and therefore the piecewise regression was set up to establish four knots. TTmin was then set at the corresponding point in time. A Shapiro-Wilks test was selected due to the small sample size to ensure all data were normally distributed. Statistical computations were performed using Microsoft Excel for Windows (Version 16.0.4738.1000) and MathWorks Matlab for Windows (Version 9.3.0.713570 R2017b). For the purpose of this paper the dominant hand was used for statistical analysis, as the dominant hand in comparison to non-dominant hand in climbers shows significant variation in regard to oxygenation kinetics^28^.

## Results

**Figure 2:**
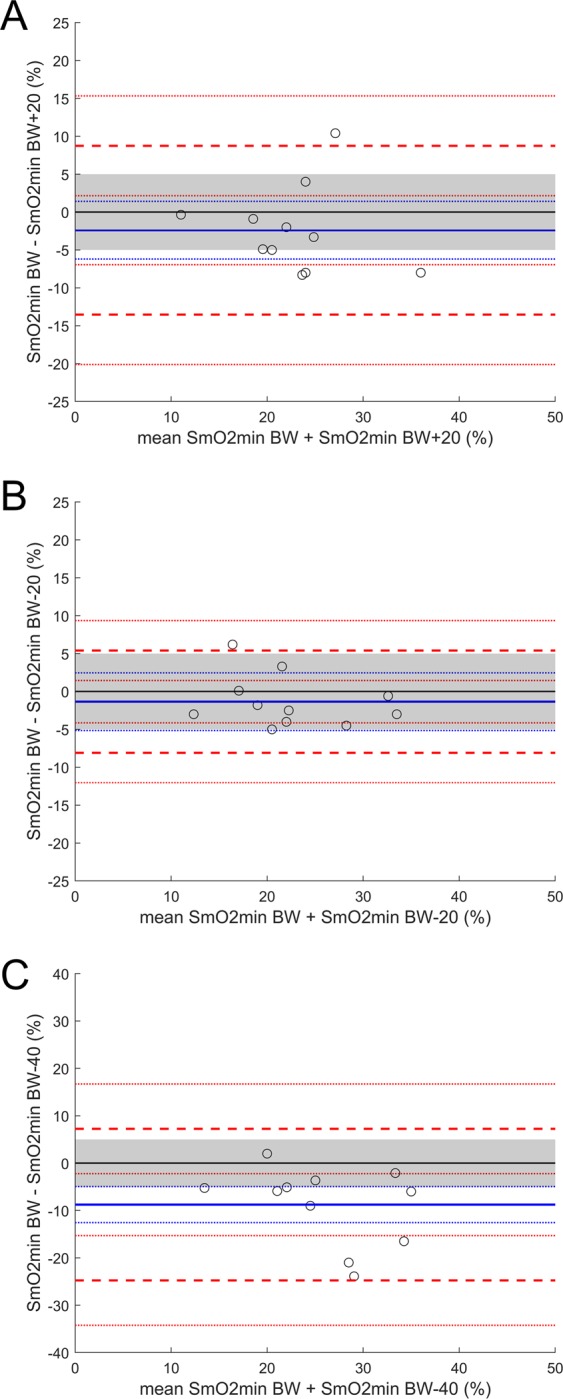
Bland-Altman plot looking at agreement between SmO_2_min during the four trial conditions with a priori equivalency interval (EI) set at  ±5% (shaded area). The solid blue line identifies the mean bias (MB) and the dotted blue lines the 95% CI. The dashed red lines identify the upper and lower limits of agreement at ±1.96  SD with 95% CI. (**A**) *M*_*BW-BW* + 20_ = −2.4%, 95% CI [1.4, −6.2]; (**B**) *M*_*BW-Bw−20*_ = −1.3, 95% CI [2.5, −5.1]; (**C**) *M*_*BW-BW −40*_ = −8.8%, 95% CI [−5.0, −12.6].

The four test conditions resulted in the expected force-duration relationship predicted by the study design (Table 1). Evaluating the graphical output of the Bland-Altman analysis for SmO_2_min (Fig. 2) at high intensity a consistent SmO_2_min is reached regardless of duration of the finger-hang. An acceptable agreement can be discerned in both BW +20 and BW −20 conditions against the BW condition. Both comparisons show no systemic bias against the line of equality indicating no statistical difference, and well bounded upper and lower limits of agreement to the a priori determined EI. In both cases, however statistical equivalency can not be clearly discerned as CI expand beyond the the a priori set EI.

**Table 1:** Mean (±SD) for minimally attainable muscle oxygenation (SmO_2_min), time to SmO_2_min (TTmin) and time to failure (TTF) during the four experimental conditions. For statistical analysis see text.

	Trial 1: BW	Trial 1: BW +20	Trial 1: BW −20	Trial 1: BW −40
SmO_2_min (%)	21.6 ± 6.4	24.0 ± 7.0	23.0 ± 7.3	30.4 ± 9.1
TTmin (s)	66.9 ± 20.8	39.7 ± 12.8	67.5 ± 33.1	112.6 ± 107.8
TTF (s)	77.5 ± 27.8	47.7 ± 12.4	106.4 ± 24.5	311.5 ± 209.0

**Figure 3:**
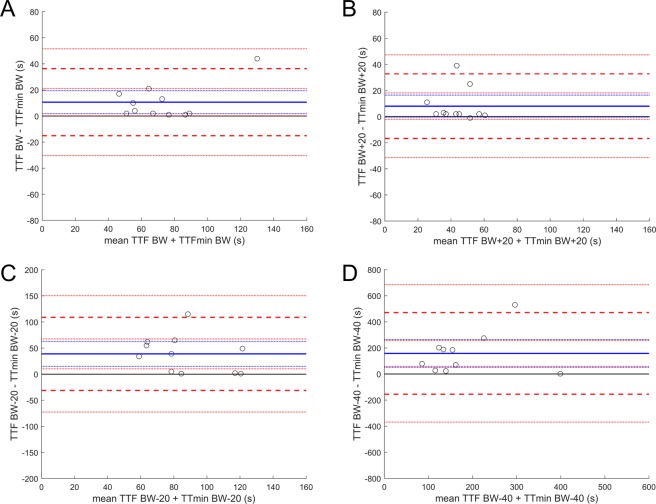
Bland-Altman plot looking at agreement between TTF and TTmin during the four trial conditions. The solid blue line identifies the mean bias (MB) and the dashed blue lines the 95% CI. The dashed red lines identify the upper and lower limits of agreement at ±1.96  SD with 95% CI. (**A**) *M*_*BW TTF-BW TTmin*_ = 10.6 s, 95% CI [19.4, 1.9]; (**B**) *M*_*BW* + 20 *TTF-BW* + 20 *TTmin*_ = 8.0 s, 95% CI [16.5, −0.5]; (**C**) *M*_*BW−20 TTF-BW−20 TTmin*_ = 38.9 s, 95% CI [62.8, 15.0]; (**D**) *M*_*BW −40 TTF-BW −40 TTmin*_ = 158.1 s, 95% CI [265.0, 51.2].

For the BW −40 condition against the BW condition there was no statistical equivalency and a clear difference, with a systemic bias towards higher SmO_2_min values for the BW −40 condition (Fig. 2). As with SmO_2_min, for the high intensity trials, TTF and TTmin are comparable indicating SmO_2_min capacity and rate of depletion as a potential predictor of task failure. Comparison between BW and BW +20 (Fig. 3) show a good degree of agreement. These two conditions show no clear systemic bias from the line of equality and should not be considered statistically different, while a claim of equality remains absent. In both cases upper and lower limits of agreement remain well bounded with a tendency to greater values for TTF. While BW −40% condition to a much greater extent than the BW −20% condition, both are statistically different and not equivalent (Fig. 3). This illustrates, when comparing all conditions, a clear bias towards increasing TTF vs. TTmin with increasing intensity.

## Discussion

The results of this study show that for the three high intensity conditions, isometric muscle contraction results in the same SmO_2_min value at the point of failure. These results can be further extrapolated to confirm the relationship between TTF and TTmin, which also show good agreement. Considering these findings, a value as simple as SmO_2_min and rate of SmO_2_ change can be used to potentially predict failure during isometric muscle contraction. Similar results were reported during sustained static gripping tasks^16^, where maximum and minimum values for deoxyhemoglobin and oxyhemoglobin respectively were correlated to loss of force production.

In order to maintain isometric contraction a continuous utilization of energy stores must take place. This ATP breakdown is buffered by high energy phosphate transfer from PCr to ADP. Evidence supports that PCr is not only reconstituted via oxidative means but dependent on oxygen availability^32,33^. Considering the importance of oxidative capacity on energy flux in contracting muscles it stands to reason that muscle oxygenation as measured by NIRS is a suitable tool to assess and predict muscle contraction failure for a given task. This argument is further enhanced by the relationship between muscle oxidative capacity as measured by P-MRS and NIRS. For this reason, a plethora of studies have demonstrated the diagnostic qualities of NIRS, from threshold testing^34,35^, muscle damage analysis^36^ and high intensity interval response^37,38^. Similar physiological reasoning has been applied to NIRS measurements during sport climbing, highlighting the importance of specific NIRS derived parameters^17^. SmO_2_min as an indicator of muscle oxidative capacity and a predictor of performance for climbing specifically is discussed by both Balas *et al*.^12^ and Fryer *et al*.^13^. However, in a recent publication Balas *et al*.^17^ moves forward with caution in applying SmO_2_min as a reliable marker as in their findings it had a low reliability and large variation. It is important to note that Balas *et al*.^17^ identify the parameter as Tissue Saturation Index (TSI) minimum rather than SmO_2_min, which already may go a long way in clarifying the contradictory results. The results of this study show a consistency in SmO_2_min at high intensity efforts. The NIRS device used in this experiment presumes to isolate the muscle tissue through an a priori Monte Carlo modelling for different tissue properties and photon path-length variations using the four wavelengths identified^21^. In this way, variations such as cutaneous and subcutaneous blood flow, as well as dilution from low metabolically active signals can be minimized and the resulting parameter is called SmO_2_. The device used by Balas *et al*.^17^ assumes homogeneity and infinite tissue and therefore does not distinguish between muscle tissue, adipose and skin resulting in the parameter TSI^22^. This discrepancy may be the reason for the discussion at hand. Here it is important to note that there is no consensus gold standard for SmO_2_ or TSI measures.

While for the high intensity conditions the hypothesis holds true, it is evidently clear that at lower intensities the paradigm fails to be applicable. This is potentially caused by a multitude of factors. The first which needs to be further evaluated is a study design limitation, in that performance was controlled externally, but not effectively measured. Considering the experimental population, if the task is set at merely maintaining hand position and not falling, the ability to release tension between the left and right hand could explain the volatility in the NIRS data at low intensity. Both sustained static gripping^16^ and in repeated hand grip activity^14^ showed changes in NIRS signal with decreases in performance output; therefore, it is safe to assume that if performance output does not remain constant SmO_2_ dynamics will vary. It is almost certain that at low intensities force exerted on the wooden rung was not constant between left and right hand. Tightly linked to the first comment, lower contraction force or changing contraction force offers the increased opportunity for reperfusion. Numerous studies discuss the effect of blood flow and blood volume changes on the NIRS signal and its confounding effects^39,40^. It is highly likely that in comparison to the higher intensity bouts, the low intensity bouts allow for a greater degree or chance of muscle reperfusion. More investigation is needed into the ability to predict performance at lower and varying intensity work rates using NIRS.

The ability to evaluate task success and failure using NIRS, during sport specific applications, could provide training and competition insight. It could provide trainers and athletes pacing strategies or rest planning strategies. Furthermore, it could provide alternative metrics to coordinate trainings which include interval type sessions. While more applied research is needed to find the true cost-benefit analysis of these tools, it is a conceivable path forward.

## Conclusion

A growing body of research supports the reliable application of NIRS to assess performance in sport climbing^13,17,41,42^. NIRS specific parameters can reasonably be applied to training processes and performance diagnostics to further develop athletes and educate coaches on physiological processes. While it is not clear which dynamics or specific parameters will be most beneficial in terms of diagnostics or training coordination it is clear that a relationship exists. In the specific case of this investigation it would appear that simply understanding the limit of SmO_2_ available to an athlete an a priori *in situ* prediction could be made with a certain degree of confidence at high intensity efforts. This can then be applied as mentioned to training coordination and diagnostics, or perhaps even in-field or in-event failure predictions.
